# Exercise Prescription for Falls Prevention in Older Adults: A Before–After Study of an Online Educational Course for Fitness Instructors

**DOI:** 10.1155/jare/2427862

**Published:** 2026-10-04

**Authors:** Jess M. Hennell, Dina Pogrebnoy, Alexandra Gorelik, Romi Haas

**Affiliations:** ^1^ Department of Physiotherapy School of Primary and Allied Health Care Faculty of Medicine Nursing and Health Sciences Monash University Melbourne Australia monash.ac.za; ^2^ Department of Physiotherapy Peter James Centre, Eastern Health Melbourne Australia; ^3^ School of Health Sciences University of Newcastle Callaghan New South Wales Australia newcastle.edu.au; ^4^ Department of Physiotherapy Western Health Melbourne Australia westernhealth.org.au; ^5^ Musculoskeletal Health Unit and Wiser Health Care Group School of Public Health & Preventive Medicine Monash University Melbourne Australia monash.ac.za

**Keywords:** exercise prescription, falls prevention, fitness instructors, older adults, online education

## Abstract

**Purpose:**

Exercise reduces falls in older adults, yet fitness instructors may lack formal education in prescribing effective fall prevention exercises. This study evaluated the quality of strength and balance exercises prescribed by fitness instructors before and after completing an online course on exercise prescription for falls prevention in older adults.

**Materials and Methods:**

In a before–after study, fitness instructors completed an exercise prescription task based on a clinical scenario immediately before and after participation in an online educational course. On each occasion, participants prescribed one strength and one balance exercise. Exercises were assessed using a rubric informed by evidence‐based guidelines. Pre–post changes in exercise quality were analyzed using paired *t*‐tests for strength and balance exercises separately.

**Results:**

Seventy‐seven fitness instructors completed the course, with 55 included in the analysis. Participants were predominantly female (86%), with a mean age of 51 years (SD 12.6) and a median of 12 years since qualification (IQR 7–23). Exercise quality improved by 9.1% (97.5% CI 6.6–11.6) for strength and 7.3% (97.5% CI 3.5–11.0) for balance exercises.

**Conclusions:**

An online educational course was associated with improved exercise prescription quality, although the magnitude and educational significance of these improvements remain uncertain. Further research is needed to confirm these findings and determine whether improvements in exercise prescription translate to changes in practice and improved outcomes for older adults.

## 1. Introduction

Falls pose a serious risk for older adults, with a quarter of those aged over 65 years in the community experiencing at least one fall each year [1]. Among those who fall, around 40% will experience multiple falls within the same year [2, 3]. Globally, falls rank as the eighth leading cause of disability in adults aged 75 years and older [4]. In Australia alone, falls led to approximately 133,000 hospitalizations, 5000 deaths, and an estimated AUD $2.3 billion in healthcare costs in 2019 [5].

There is high‐certainty evidence that exercise is effective in reducing falls. A large systematic review including 116 trials with 25,160 participants found that exercise reduced the rate of falls by 23% in community‐dwelling older adults [6]. To be effective, exercise programs should include exercises that challenge balance and are delivered at an adequate dose in terms of frequency, intensity, and duration [7]. Guidelines recommend exercise programs targeting functional balance and strength training should be offered to all older adults regardless of their fall risk [8, 9]. These programs should be ongoing in nature and be delivered by appropriately trained professionals such as physiotherapists, exercise physiologists, and trained exercise professionals who can tailor exercises according to an individual’s functional status and comorbidities [8]. Prescribing exercises for falls prevention requires complex clinical decision‐making, including the selection and progression of exercises tailored to an individual’s functional status and risk profile [10].

Previous studies have demonstrated that exercise programs for older adults can be effectively delivered by trained nonphysiotherapy providers, including peer mentors and community‐based instructors, highlighting the potential to build capacity within broader exercise workforces [11–14]. As the global demand for fitness programs tailored to older adults increases, fitness instructors (including personal trainers, gym instructors, and group exercise instructors) are well positioned to support exercise programs for this population. However, the capability of this workforce to independently prescribe evidence‐based strength and balance exercises for falls prevention has not been established. In Australia, a Certificate IV in Fitness provides dedicated units on strength training but does not offer equivalent formal training for balance exercise prescription [15]. Building capability within this large, community‐based workforce could improve access to evidence‐based falls prevention programs; however, qualitative evidence suggests that fitness instructors require additional training and support to prescribe balance exercises effectively [16].

Completing a short educational course may support fitness instructors to further develop the skills required to prescribe evidence‐based exercises for falls prevention. Although prior research has shown that additional education can improve exercise prescription knowledge for falls prevention among health professionals and exercise science university students [17, 18], its impact on fitness instructors remains unclear. To address this gap, we adapted an online course on exercise prescription for fall prevention specifically for fitness instructors. The primary aim of this study was to evaluate the quality of exercises prescribed by fitness instructors to older adults at risk of falls before and after completing this online educational course. Secondary aims were to examine differences in the quality of strength versus balance exercises prescribed and to compare outcomes between two consecutive course cohorts in 2018 and 2020.

## 2. Materials and Methods

### 2.1. Study Design

This study used a retrospective before‐and‐after (pre–post) intervention design to evaluate the impact of an online educational course on exercise prescription for falls prevention in older adults. As there are no specific reporting guidelines for before‐and‐after studies, this study was reported in accordance with the STrengthening the Reporting of OBservational studies in Epidemiology (STROBE) checklist [19]. Ethical approval was obtained from the Monash University Human Research Ethics Committee (reference number 27179). A waiver of consent was granted, as the data were routinely collected and stored in a de‐identified form, making it impractical to obtain informed consent from individual participants.

### 2.2. Participants

Eligible participants were fitness instructors (including personal trainers, gym instructors, or group exercise instructors) who enrolled in the online course titled “Exercise Prescription for Falls Prevention” in either of two consecutive cohorts during April 2018 and May 2020. Those trained as exercise physiologists, allied health assistants, or health professionals (e.g., physiotherapists) were excluded from the study, as the course content was specifically tailored to address the needs of fitness instructors. The Community Based Health Policy & Programs Branch of the Victorian Government Department of Health and Human Services subsidized 80% of the registration fee for eligible Victorian applicants with a background in fitness instruction.

### 2.3. Intervention

The “Exercise Prescription for Falls Prevention” short course was initially developed in 2010 to upskill clinicians with a bachelor’s degree in any health science in the area of exercise prescription for falls prevention. In response to participant feedback and demand, the online course was adapted in 2018 to address the specific needs of fitness instructors. A randomized trial and economic analysis found that the online delivery format was as effective as face‐to‐face in terms of participant satisfaction, knowledge acquisition, and behavior change, while also being more cost‐efficient for education providers [20, 21]. The revised curriculum aimed to equip participants with the knowledge and skills to prescribe evidence‐based exercises for falls prevention. The course content covered the physiological principles of exercise prescription for falls prevention, exercise selection and progression, assessment procedures for balance and mobility, and strategies to enhance adherence and support behavioral change. Further details of the online course are provided according to the Template for Intervention Description and Replication (TIDieR) guidelines (Supporting File 1) [22] and the 4Ps model of quality in education design (Supporting File 2) [23].

### 2.4. Measurement

Course participants were required to prescribe two exercises (one targeting balance and one targeting lower limb strength) for a 79‐year‐old female with a history of two falls in the past two months (Supporting File 3). This task was completed before and after engaging with the course material to evaluate the change in exercise quality and application of knowledge.

A rubric was developed to assess the quality of each prescribed exercise (Supporting File 4). This was based on key exercise prescription principles, including specificity, overload, progression, and FITT (Frequency, Intensity, Time, Type) [24, 25], as well as best practice recommendations for balance [6, 7] and strength training [26]. The rating criteria included goal of exercise, frequency/time, intensity, type, progression, safety, and clarity of instructions. Frequency (days per week) and time (repetitions and sets) were combined, as both contribute to achieving the recommended dosage (e.g., 2 h per week for balance training).

Two physiotherapists with expertise in exercise prescription for falls prevention piloted the rubric using two different exercises for both strength and balance. Modifications were made to improve clarity and consistency. These included specifying dosage recommendations for balance exercises, adding an extra scoring category for exercise progression, and refining the type of exercise to better suit the clinical scenario. The agreement between assessors improved from 75% to 86% after these modifications. The same physiotherapists independently rated each prescribed exercise using the prespecified rubric and were aware of whether prescriptions were completed before or after course participation. Pre‐ and postcourse prescriptions were rated separately and linked only after rating to calculate changes. Disagreements were resolved through discussion until consensus was reached.

### 2.5. Data Analysis

Participant demographic characteristics were summarized using descriptive statistics. Categorical variables (gender, highest relevant qualification, geographical location, and workplace) were presented as frequency (N) and percentage (%). Continuous variables were described using means and standard deviations (SDs) for age and medians and interquartile ranges (IQRs) for years since qualification.

Differences in baseline characteristics between fitness instructors who completed both the pre‐ and postcourse exercise prescription tasks and those who did not were assessed using independent *t*‐tests for age, Mann–Whitney *U*‐tests for years since qualification, and Fisher’s exact tests for all categorical variables. The same statistical tests were used to compare baseline characteristics between the 2018 and 2020 cohorts.

Each exercise was scored using an 18‐point rubric and converted to a percentage. The distribution of rubric scores was examined for normality using histograms and the Shapiro–Wilk test. Pre–post differences in exercise quality were evaluated using paired *t*‐tests, conducted separately for strength and balance exercises. In the absence of an established threshold for a minimally important change for exercise prescription quality, an improvement of 10% or more was deemed a priori to be “educationally” meaningful. A sensitivity analysis was conducted to assess the effect of including participants who made no changes to their prescribed exercises on the estimated change in exercise prescription quality.

To address our secondary aim of comparing strength versus balance exercise prescription quality, paired *t*‐tests were conducted to compare (a) precourse strength versus balance exercises; (b) postcourse strength versus balance exercises; and (c) the change scores for strength versus balance exercises within participants. To compare outcomes between the 2018 and 2020 cohorts, independent samples *t*‐tests were used to compare mean change scores between cohorts, conducted separately for strength and balance exercises.

A post hoc descriptive analysis was conducted to explore patterns of change across rubric domains and differences between strength and balance exercise prescriptions. For each domain, the percentage of participants who prescription improved, remained unchanged, or regressed from pre‐ to postcourse were calculated separately for strength and balance exercises.

All data analyses were performed using Stata Version 15 (STATA Corp LLC, College Station, TX, USA). Strength and balance exercise quality were specified a priori as co‐primary outcomes, as both domains are necessary to align with evidence‐based exercise prescription principles for fall reduction. To account for multiplicity, a Bonferroni‐adjusted two‐sided significance level of *p* < 0.025 was applied for all analyses, with corresponding 97.5% confidence intervals reported.

### 2.6. Sample Size Calculation

A minimum of 20 participants was required to detect an absolute change of at least 10% in exercise prescription quality, based on a baseline score of 60% ± 12.4%, a two‐sided alpha level of 0.025 to adjust for multiplicity, and 80% power. In our previous trial with health professionals, median postcourse scores were 78.6% (IQR 68.5–85.1) for the online group and 78.6% (IQR 70.8–86.9) for the face‐to‐face group [20]. We expected lower baseline and overall scores in our group of fitness instructors.

## 3. Results

### 3.1. Participant Characteristics

Figure 1 outlines participant enrollment and eligibility, course completion, and reasons for exclusion. A total of 118 participants enrolled in the course, of whom 106 were fitness instructors. Seventy‐seven participants (73%) successfully completed the course requirements, and of these, 55 (71%) were included in the study (34 from 2018, 21 from 2020).

**FIGURE 1:**
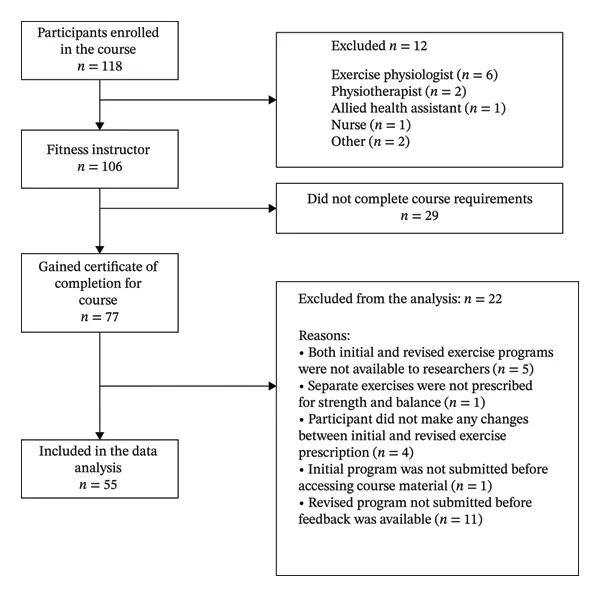
Participant flowchart including enrollment, eligibility, course completion, and reasons for exclusion.

Participant demographic and professional characteristics are summarized in Table 1. Eighty‐six percent (*n* = 47) were female, with a mean age of 51 years (SD 12.6). Seventy‐six percent (*n* = 41) worked in metropolitan locations. The median time since qualification was 12 years (IQR 7–23). Most participants held a Certificate IV (*n* = 34, 62%) or III (*n* = 8, 15%) as their highest relevant qualification. The most common workplaces were community or council leisure centers (*n* = 22, 40%), self‐employed businesses (*n* = 13, 24%), and gym or personal training studios (*n* = 9, 16%), with 11% (*n* = 6) working across multiple settings.

**TABLE 1:** Demographic and professional characteristics of fitness instructors included and excluded from the study, by course cohort.

Characteristic	Included in the study (*n* = 55)	Excluded from the study (*n* = 22)	2018 cohort (*n* = 34)	2020 cohort (*n* = 21)
Female, *n* (%)	47 (86)	14 (64)	26 (77)	21 (100)
Age, mean (SD)	50.9 (12.6)	48.3 (11.4)	51.6 (12.6)	49.8 (12.7)
Location^#^, *n* (%)				
Metropolitan	41 (75)	18 (82)	27 (79)	14 (67)
Regional	5 (9)	0 (0)	2 (6)	3 (14)
Rural	8 (14)	4 (18)	5 (15)	3 (14)
(Missing)	1 (2)	0 (0)	0 (0)	1 (5)
Years since qualification, median (IQR)	12 (7–23)	5 (3–15)	12 (7–18)	14 (8–24)
Highest relevant qualification, *n* (%)				
Certificate III	8 (15)	1 (4)	3 (9)	5 (24)
Certificate IV	34 (62)	10 (45)	20 (59)	14 (67)
Diploma	1 (2)	3 (14)	1 (3)	0 (0)
Bachelor or higher	3 (5)	3 (14)	1 (3)	2 (9)
(Missing)	9 (16)	5 (23)	9 (26)	0 (0)
Workplace^∗^, *n* (%)				
Community/council leisure center	22 (40)	7 (32)	12 (35)	10 (48)
Gym/personal training studio	9 (16)	8 (36)	3 (9)	6 (29)
Private practice	2 (4)	1 (5)	1 (3)	1 (5)
Aged care	3 (5)	1 (5)	2 (6)	1 (5)
Health service	3 (5)	1 (5)	2 (6)	1 (5)
Community health	2 (4)	2 (9)	2 (6)	0 (0)
Self employed	13 (24)	2 (9)	12 (35)	1 (5)
Other	7 (13)	2 (9)	2 (6)	5 (24)

^#^Defined by the Monash Medical Model.

^∗^The overall % is > 100%, as some participants had multiple workplaces.

Demographic and professional characteristics were similar between fitness instructors who completed the course requirements and those who did not, except that the participants who completed the course were more experienced (median 12 vs. 5 years). Demographics were generally comparable between cohorts, although the 2020 cohort comprised only female participants, compared with 77% female in 2018.

### 3.2. Pre‐ and Postcourse Exercise Prescription Quality

Both strength and balance exercise prescription quality improved following completion of the online educational course (Table 2). For strength exercises, the mean score increased from 63.2% (SD 16) precourse to 72.3% (SD 18.2) postcourse, with a mean improvement of 9.1% (97.5% CI 6.6–11.6). For balance exercises, the mean score rose from 57.1% (SD 14.4) precourse to 64.3% (SD 12.6) postcourse, with a mean improvement of 7.3% (97.5% CI 3.5–11.0). The 97.5% confidence intervals are reported to account for multiplicity across the two primary outcomes.

**TABLE 2:** Strength and balance exercise quality before and after participation in the online course.

Exercise type	Precourse mean % (SD) *n* = 55	Postcourse mean % (SD) *n* = 55	Mean change % (97.5% CI)
Strength	63.2 (16.0)	72.3 (18.2)	9.1 (6.6–11.6)
Balance	57.1 (14.4)	64.3 (12.6)	7.3 (3.5–11.0)

A sensitivity analysis including the four participants whose exercise prescriptions were unchanged yielded similar overall results, although the estimated improvements were lower. For strength exercises, the mean score rose from 61.4% (SD 17.9) precourse to 69.9% (SD 20.6) postcourse, with a mean improvement of 8.5% (97.5% CI 6.0–10.9). For balance exercises, the mean score rose from 55.5% (SD 16.1) precourse to 62.2% (SD 17.3) postcourse, with a mean improvement of 6.8% (97.5% CI 3.2–10.3) (Supporting File 5).

### 3.3. Within‐Participant Comparison of Strength vs. Balance Exercise Quality

The quality of strength exercises prescribed was higher than that of balance exercises both precourse (mean difference 6.1%) and postcourse (mean difference 8.0%) (data reported in Table 2). However, the mean improvement in quality did not differ between strength (9.1%, 97.5% CI 6.6–11.6) and balance exercises (7.3%, 97.5% CI 3.5–11.0), with a within‐participant difference of 1.8% (97.5% CI −1.2 to 4.8), suggesting comparable improvements for both exercise types.

A post hoc descriptive analysis of individual rubric domains identified different patterns of change for strength and balance exercises (Supporting File 6). For strength exercises, improvements were most frequently observed for frequency/time (52.7%), followed by progression (34.5%), with little regression in either domain (5.5% and 0%, respectively). For balance exercises, improvements were most frequently observed for safety (41.8%) and frequency/time (41.8%), with relatively little regression in these domains (1.8% for safety and 3.6% for frequency/time). Changes in intensity, progression, and type were more mixed, with 7.3% improving and 14.5% regressing for intensity, 23.6% improving and 14.5% regressing for progression, and 16.4% improving and 9.1% regressing for type.

### 3.4. Comparison Between 2018 and 2020 Course Cohorts

Improvements in exercise prescription quality were similar between the 2018 and 2020 course cohorts (Table 3). For strength exercises, mean change was 9.6% (97.5% CI 6.6–12.7) in 2018 and 8.2% (97.5% CI 3.5–12.9) in 2020, with a between‐cohort difference of −1.4% (95% CI −6.7 to 3.9). For balance exercises, mean change was 7.7% (97.5% CI 2.3–13.0) in 2018 and 6.6% (97.5% CI 1.3–11.9) in 2020, with a between‐cohort difference of −1.1% (97.5% CI −8.4 to 6.2).

**TABLE 3:** Improvements in strength and balance exercise quality by course cohort (2018 versus 2020).

Exercise type	Cohort	Precourse mean (SD)	Postcourse mean (SD)	Mean change (97.5% CI)	Between‐cohort difference % (97.5% CI)
Strength	2018	62.9 (19.1)	72.5 (21.8)	9.6 (6.6–12.7)	
	2020	63.8 (9.2)	72.0 (10.2)	8.2 (3.5–12.9)	−1.4 (−6.7 to 3.9)

Balance	2018	56.0 (15.3)	63.7 (13.6)	7.7 (2.3–13.0)	
	2020	58.7 (12.9)	65.3 (11.1)	6.6 (1.3–11.9)	−1.1 (−8.4 to 6.2)

*Note: n* = 34 for the 2018 cohort, *n* = 21 for the 2020 cohort. Mean change and between‐cohort differences are reported as absolute percentage point improvements. 97.5% confidence intervals reflect a conservative significance threshold of *p* < 0.025 applied to all analyses.

## 4. Discussion

Participation in the online educational course was associated with improved quality of strength and balance exercises prescribed by fitness instructors, with absolute gains of 9.1% and 7.3%, respectively. Sensitivity analyses including participants with unchanged prescriptions yielded lower estimates of improvement. Although strength exercises were of higher quality than balance exercises before (6.1%) and after course participation (8.0%), the magnitude of improvement (1.8%) was comparable between the two. Improvements were consistent across both the 2018 and 2020 cohorts. However, the observed improvements and the difference in quality between strength and balance exercises did not exceed our prespecified threshold of a 10% difference to indicate an educationally meaningful change. Taken together, these findings suggest that, while differences in exercise prescription quality were observed, the magnitude of these improvements may be modest, and their educational significance remains uncertain.

Despite the modest magnitude of these improvements, the findings are consistent with previous studies demonstrating that educational programs can improve knowledge of exercise prescription for falls prevention among health professionals and exercise science university students. For example, an Australian randomized controlled trial found that a one‐day face‐to‐face workshop improved both knowledge and self‐reported exercise prescription behavior in health professionals, primarily physiotherapists [18]. Similarly, a before‐and‐after study, also conducted in Australia, demonstrated that a 13‐week university‐based falls prevention education program improved knowledge and confidence in exercise science students [17]. Extending this work, the current study demonstrates that fitness instructors, who typically do not have university‐level qualifications, can also benefit from targeted education. These improvements were demonstrated using a practical exercise prescription task based on a clinical scenario, rather than theoretical knowledge alone.

Our findings build on previous evidence regarding the role of nonhealth professionals in delivering falls prevention exercise. Previous research demonstrates that falls prevention exercise programs can be effectively delivered by nonhealth professionals (including trained physical educators, community‐based exercise leaders and personal trainers) as well as health professionals [6, 13, 14]. However, in these models, exercise providers were trained to deliver established exercise programs, with exercise selection provided to guide their delivery [13, 14]. By demonstrating improved ability to independently prescribe evidence‐based exercises for falls prevention, our study extends this literature to fitness instructors. For older adults at higher risk of falls or with complex comorbidities who require comprehensive falls risk assessment, the clinical expertise of health professionals may be advantageous to ensure safety and appropriate individualization [8, 27].

The strength exercises prescribed were of consistently higher quality than balance exercises. This may reflect greater exposure of fitness instructors to resistance training in formal fitness qualifications, which include dedicated units on strength training but lack equivalent content on balance training [15]. Similarly, AUSactive, the Australian national industry association for exercise professionals, provides guidelines for resistance training, but no equivalent guidance exists for balance training [28]. The domain‐level findings provide further insight into this difference, with improvements most commonly observed in frequency/time for both strength and balance exercises followed by safety for balance exercises and progression for strength exercises. However, greater variability was observed across some balance domains, particularly intensity, where regression was more common than improvement, suggesting an area for further emphasis in future education. Incorporating evidence‐based content on balance training into foundational fitness curricula and developing specific guidelines for balance exercise prescription may improve the quality of balance exercises delivered by fitness instructors.

In addition to the content of the education, the online format has implications for the accessibility and scalability of falls prevention training. A key feature of our falls prevention educational course was its delivery online, without any face‐to‐face component. This mode of delivery offers a flexible and scalable alternative to traditional in‐person training. Similar to our findings, a prospective pre–post quasi‐experimental study conducted in Australia found that an e‐learning falls prevention course for physiotherapists was feasible, acceptable, and improved knowledge test scores [29]. Online education is increasingly recognized as a cost‐effective and accessible method for teaching clinical reasoning skills to health professionals [30, 31], and our findings extend this evidence to fitness instructors by demonstrating improvements in applied knowledge of exercise prescription for falls prevention.

The findings across the 2018 and 2020 cohorts provide further insight into the applicability of the course across different groups of exercise instructors, course facilitators, and contexts. The 2020 cohort completed the course during COVID‐19 “stay at home” restrictions, which prevented onsite work. Despite this, participants demonstrated comparable improvements in exercise prescription quality, indicating improved applied knowledge of exercise prescription for falls prevention based on a clinical vignette, even when participants were unable to immediately apply this knowledge in the workplace. However, this study does not establish whether improvements in exercise prescription quality translated into changes in workplace practice.

A key strength of this study was the use of a practical exercise prescription task based on a clinical scenario/vignette, which allowed assessment of applied knowledge and real‐world decision‐making, an established method for evaluating clinical practice that accurately assesses the quality of care delivered [32]. This study also has several limitations. The nonrandomized pre–post design limits the ability to control for confounding variables. The assessment rubric, while informed by evidence, was not formally validated, which may affect the consistency and reliability of scoring. Raters were not blinded to the pre/post status of the exercise prescription tasks, which may have introduced assessor bias, although pre‐ and postcourse exercises were rated independently using the same prespecified rubric. Analyses were limited to participants who completed all course tasks, potentially introducing selection bias by favoring individuals who were more motivated or engaged. Participants who completed the course requirements were also more experienced than those who did not, making the findings less generalizable to less experienced fitness instructors. The clinical importance of the observed improvements in exercise prescription quality remains uncertain, including whether these changes translate to meaningful clinical outcomes for older adults. Additionally, as the study was conducted in Australia, the findings may not be generalizable to other countries.

Further research is required to determine what constitutes a meaningful difference in exercise prescription quality, whether educational gains translate to changes in real‐world practice, and ultimately whether these changes contribute to fall reductions. Formal validation of the rubric used to assess the quality of each prescribed exercise should also be prioritized, including consideration of the relative weighting of domains such as safety and instruction. Randomized controlled trials comparing targeted exercise prescription education with general falls prevention education (placebo education) could provide more definitive evidence. There is also a need for expert consensus on reporting guidelines specific to before‐and‐after study designs of educational interventions [33].

## 5. Conclusion

An online educational course on exercise prescription for falls prevention may improve the quality of strength and balance exercises prescribed by fitness instructors for older adults in the community. However, the magnitude and educational significance of these improvements remain uncertain. Further research using more rigorous study designs, such as randomized controlled trials, is needed to confirm these findings and determine whether improvements in exercise prescription translate into changes in workplace practice and ultimately reduced falls risk.

## Funding

This study was supported through funding from the Community Based Health Policy & Programs Branch of the Victorian Government Department of Health & Human Services.

Open access publishing was facilitated by Monash University, as part of the Wiley ‐ Monash University agreement via the Council of Australasian University Librarians.

## Conflicts of Interest

The authors declare no conflicts of interest.

## Supporting Information

Additional supporting information can be found online in the Supporting Information section.

## Supporting information


**Supporting Information** Supporting File 1. Intervention description using the Template for Intervention Description and Replication (TIDieR) checklist. Supporting File 2. Modified 4Ps model of quality in education design. Supporting File 3. Clinical scenario. Supporting File 4. Scoring rubric for evaluating the quality of strength and balance exercises prescribed by course participants. Supporting File 5. Sensitivity analyses of pre‐to postcourse changes in strength and balance exercise prescription quality. Supporting File 6. Percentage of participants who exercised prescription improved, remained unchanged, or regressed across rubric domains after participation in the online course.

## Data Availability

The data that support the findings of this study are available on request from the corresponding author. The data are not publicly available due to privacy or ethical restrictions.
