# Supplementary material for: Exercise Prescription for Falls Prevention in Older Adults: A Before–After Study of an Online Educational Course for Fitness Instructors
Source: J Aging Res. 2026 Oct 4;2026:2427862. doi: 10.1155/jare/2427862 (PMC13635795; doi:10.1155/jare/2427862)
Supplement: Supplementary file 1 [file JARE-2026-2427862-s001.docx]

**Supplementary File 1. Intervention description using the Template for Intervention Description and Replication (TIDieR) checklist**

| **TIDieR Item** | **Details** |
| --- | --- |
| 1. Name of intervention | Exercise prescription for falls prevention online course |
| 2. Why | This course was developed by leading falls prevention researchers and clinicians in response to an identified evidence-practice gap that exercise prescribers were not prescribing the right type of exercise or the correct dose to optimise the known falls prevention benefit. The purpose of the course was to upskill participants to prescribe exercises in-line with evidence-based best practice recommendations.  The content of the course was informed by three scoping activities: a review of the falls-prevention program literature to establish common elements in existing falls-education programs; phone interviews conducted with 24 clinicians who were leaders in falls prevention to establish current practice in specialized clinics (Haas et al., 2012); and phone interviews conducted with six target audience representatives from multiple professions to identify the knowledge gap between expert clinicians and the education target audience. The course content then underwent an external review by a falls-prevention researcher, a clinical leader, an academic leader, and a member of the falls-risk target audience. All learning objectives for both interventions were mapped and linked to relevant resources and tasks and were matched in content and time requirements. |
| 3. Materials | Information was provided in the form of:   - Videos (less than 10 minutes each) - Written information - Optional further reading links including research articles and guidelines for falls prevention programs such as Otago exercise program (John et al., 2003).   Interactive learning with peers was conducted via discussion boards. Course facilitators provided feedback and guidance to participants.  Eleven tasks were set for completion throughout the course including 3 discussion posts, 4 quizzes, 3 practical tasks and a final knowledge test. The practical tasks included a self-recorded video demonstrating the performance of a balance or mobility assessment tool on a pretend patient, exercise prescription in response to a client scenario, and motivational interviewing practice. |
| 4. Procedures | The course content is outlined below:  Week 1 - Getting started   - Introduction - Learning objectives - Muscle actions - Multifactorial nature of falls - Evidence supporting exercise prescription for falls prevention - Principles of exercise prescription   Week 2 - Exercise for falls prevention   - Considerations for the older adult - Dosage in exercise prescription - Refining your exercise selection - Making exercise fun - Exercise progression   Week 3 - Assessing balance and mobility   - Introduction to assessing balance and mobility - Sit to stand - Step test - Timed up and go - Functional reach test - Quadriceps strength - Observing movement quality - Postural sway   Week 4 - Maximising program adherence   - Maximising program adherence - Behaviour change - Models of behaviour change - Motivational interviewing - Motivational interviewing in practice - Client example - the importance of exercise - SMART goals |
| 5. Who provided | Three physiotherapists with clinical backgrounds in falls prevention actively facilitated the course.  Facilitator 1 (RH) - Involved with the development of the course and facilitated all iterations. Over 15 years relevant clinical experience including roles in falls prevention and five years relevant research experience.  Facilitator 2 (DP) - Facilitated 2018 and 2020 course, over 15 years relevant clinical experience including working in Falls and Balance clinic, teaching undergraduate physiotherapists with some research experience in falls prevention.  Facilitator 3 (JH) - Facilitated 2020 course, over ten years relevant clinical experience including work in Falls and Balance Clinic. |
| 6. How | The educational course was delivered online via the Moodle™ platform in a self-paced format. |
| 7. Where | The course was made available virtually by The Faculty of Medicine, Nursing and Health Sciences at Monash University in Melbourne, Australia. Participants were all located in Australia, with the majority in Victoria. |
| 8. When and how much | The course was available from 9th April until 6th May 2018 and from 4th May until 31st May 2020. The course was expected to take approximately 8-10 hours to complete over a 4-week time period. Additional material was supplied for participants who wished to expand on the teachings within the course. In 2018, participants reported to have spent a median of eight hours (IQR 5-11.5 hours) engaged in the core materials and an additional four hours interacting with the further reading materials. In 2020, participants reported to have spent a median of ten hours (IQR 5-11.5 hours) engaged in the core materials and an additional three hours interacting with the further reading materials. |
| 9. Tailoring | All participants had access to the same online course content. Two experienced clinicians actively facilitated the course throughout its duration and were available to answer questions and provide individualised feedback. |
| 10. Modifications | The course content underwent minor modifications during the study to update the evidence supporting exercise for falls prevention to include the most recent systematic reviews. |
| 11. How well (planned) | The educational course was implemented as planned. |
| 12. How well (actual)/ Evaluation | Participants were encouraged to complete an anonymous online evaluation with over 90% of participants reporting they were satisfied or very satisfied with both the 2018 and 2020 iterations of the course. A knowledge test was given at the completion of the course with a first attempt average of 8.1/10 in 2018 and 8.3/10 in 2020. To evaluate the effectiveness of the course in improving the application of knowledge to practice, participants were asked to complete an exercise prescription task based on a clinical scenario. The results of this task are reported in this manuscript. |

References:

Haas, R., Maloney, S., Pausenberger, E., Keating, J. L., Sims, J., Molloy, E., Jolly, B., Morgan, P., & Haines, T. (2012). Clinical decision making in exercise prescription for fall prevention. *Physical Therapy*, *92*(5), 666–679. https://doi.org/10.2522/ptj.20110130

John, C., Roberston, C., & University of Otago Medical School. (2003). *Otago exercise programme to prevent falls in older adults*. Accident Compensation Corporation.

**Supplementary File 2 - Modified 4Ps model of quality in education design**

| **Item** | **Key questions** | **Responses** |
| --- | --- | --- |
| **Presage/ Planning** | What was the rationale for the education program?  What was the purpose of the education program? | This course was developed by leading falls prevention researchers and clinicians in response to an identified evidence-practice gap that exercise prescribers were not prescribing the right type of exercise or the correct dose to optimise the known falls prevention benefit. The purpose of the course was to upskill participants to prescribe exercises in-line with evidence-based best practice recommendations. |
|  | What was the learning environment?  What resources were required to deliver the program? | The educational course was delivered online via the Moodle™ platform in a self-paced format. All participants were required to have a computer with internet access. |
|  |  |  |
|  | Who taught the education program?  How were the teachers identified/recruited?  Were the teachers qualified and/or experienced in teaching?  Were the teachers qualified and/or experienced in the topic of falls prevention (subject matter experts)? | Three physiotherapists with clinical backgrounds in falls prevention actively facilitated the course. They were recruited via their previous clinical and research work in this topic.  Facilitator 1 (RH) - Involved with the development of the course and facilitated all iterations. Over 15 years relevant clinical experience including roles in falls prevention and five years relevant research experience.  Facilitator 2 (DP) - Facilitated 2018 and 2020 course, over 15 years relevant clinical experience including working in Falls and Balance clinic, teaching undergraduate physiotherapists with some research experience in falls prevention.  Facilitator 3 (JH) - Facilitated 2020 course, over ten years relevant clinical experience including work in Falls and Balance Clinic. |
|  |  |  |
|  |  |  |
|  | Who were the learners?  What was the configuration of the audience? | Eligible participants were certified fitness instructors (including personal trainers, gym instructors or group exercise instructors) who enrolled in the online course titled ‘Exercise Prescription for Falls Prevention’ in two consecutive iterations during April 2018 and May 2020. |
|  | How many learners were educated? | 77 certified fitness instructors completed the course over the two iterations |
|  | Was an evaluation of the education program planned from the inception of the development of the program? | An evaluation strategy was embedded in the program from its inception, with participants encouraged to complete an anonymous online survey upon course completion. |
| **Process** | Were the teachers trained in how to deliver the education program?  Were the learning objectives for the education program stated?  Were learning objectives written in behavioural terms? | Facilitators underwent preparatory training to ensure familiarity with the course materials and consistency in delivering individualised feedback on course tasks.  The learning objectives for the educational course were explicitly stated, and they were written in clear behavioural terms. |
|  |  |  |
|  | Was there recognition of learners' prior knowledge?  Was there recognition of learners' prior experience? | The course was targeted at certified fitness instructors, although it did not assume or require prior knowledge or experience in prescribing exercises for falls prevention. |
|  | Were the teaching & learning methods stated?  What did the learners do in the education program? | Information was provided in the form of:   - Videos (less than 10 minutes each) - Written information - Optional further reading links   Interactive learning with peers was conducted via discussion boards.  Eleven tasks were set for completion throughout the course including 3 discussion posts, 4 quizzes, 3 practical tasks and a final knowledge test. The practical tasks included a self-recorded video demonstrating the performance of a balance or mobility assessment tool on a pretend patient, exercise prescription in response to a client scenario, and motivational interviewing practice. |
|  | What did the teachers do in the education program? | Course facilitators provided feedback and guidance to participants via the discussion boards. They also provided feedback on practical tasks and provided individualised support via email as required. |
|  | Were the teaching & learning methods constructively aligned to the learning objectives or the stated purpose of the education program? | The teaching and learning methods were constructively aligned to the learning objectives. Videos, written summaries, further reading tasks and quizzes supported knowledge of falls and exercise evidence. Practical tasks—such as assessment demonstrations, exercise planning, and motivational interviewing—enabled application of skills aligned with each objective. Peer discussions further reinforced learning and reflection. |
|  | How long did the education program take to complete? | The course was expected to take approximately 8-10 hours to complete over a 4-week period. Additional material was supplied for participants who wished to expand on the teachings within the course. In 2018, participants reported to have spent a median of eight hours (IQR 5-11.5 hours) engaged in the core materials and an additional four hours interacting with the further reading materials. In 2020, participants reported to have spent a median of ten hours (IQR 5-11.5 hours) engaged in the core materials and an additional three hours interacting with the further reading materials. |
|  |  |  |
| **Product** | What types of outcomes were evaluated?  Was there an assessment of the learners' achievement of the learning objectives or stated purpose of the education program?  Was an evaluation of the education program conducted?  How was the evaluation data gathered?  What level/s of evaluation are reported? | The education program evaluated multiple outcomes across Kirkpatrick’s training evaluation levels. Level 1 (Reaction) was assessed through an anonymous online evaluation, with over 90% of participants reporting they were satisfied or very satisfied with both the 2018 and 2020 iterations of the course. Level 2 (Learning) was assessed via a knowledge test completed at the end of the course, with first-attempt averages of 8.1/10 in 2018 and 8.3/10 in 2020. Level 3 (Behaviour/Application) was evaluated through an exercise prescription task in response to a clinical vignette, assessing participants’ ability to apply their learning in a practical scenario. |

**Supplementary File 3 – Clinical Scenario**

Elsie is a 79-year-old self-described ‘wild-child’ who has experienced two falls in the past two months – one inside, one outside. She has become anxious about her safety and mobility since she lost Tyrone, her husband. She is very fearful of falling whilst exercising in her home.

**Your assessment of Elsie has so far revealed:**

- Living independently
- Using a single point stick to get around the farm
- Self-confessed forgetfulness
- Reasonable vision
- Keeps chickens that she lets roam through her house
- Has 5 kg quadriceps strength recorded on her left side (spring gauge dynamometer) – and 17 kg in the right quadriceps strength which is within normal limits (15–29 kg)
- Dramatic increase in postural sway with removal of visual input.

**Supplementary File 4 – Scoring rubric for evaluating the quality of strength and balance exercises prescribed by course participants**

**STRENGTH EXERCISE RUBRIC**

| Item | 0 | 1 | 2 | 3 | 4 |
| --- | --- | --- | --- | --- | --- |
| *Goal* | No or unsatisfactory goal provided | Satisfactory goal provided relating to improving strength | Comprehensive goal provided relating to improving strength in given client scenario |  |  |
| *Frequency/Time* | Frequency or time not described | Frequency or time does not meet strengthening guidelines | Satisfactory frequency and/or time  (at least 2 of the following:  8-15 reps, 2-3 sets, rest period described, 2-3 times/week) | Optimal frequency and time  (8-15 reps, 2-3 sets, rest period described, 2-3 times/week) |  |
| *Intensity* | Intensity/difficulty of strength exercise not described in sufficient detail to assess appropriateness | Intensity/difficulty not appropriate for improving hypothetical client’s strength (e.g., insufficient load/resistance) | Satisfactory level of load/resistance to build strength (e.g., external weight) | Good level of load/resistance. to build strength (e.g., using body weight) |  |
| *Type* | Exercise not appropriate to improve strength | Exercise targets strength but is not appropriate for falls prevention (e.g., seated) | Functional exercise specific to client example. |  |  |
| *Progression* | No progressions provided | Inappropriate progression or insufficient detail provided | Acceptable progression(s) provided but may not be optimal choice **OR** guidance of when to progress not provided or not appropriate | Appropriate progression(s) with guidance on when to progress but room for improvement in some aspect of the progression, instructions or clarity. | Comprehensive progression(s) with qualitative guidance on when to progress  (e.g., able to complete full quantity of exercise easily with good quality of movement and without pain) |
| *Safety* | Client’s safety not considered (set up of exercise or instructions) | Satisfactory consideration of client’s safety  (set up of exercise or instructions) | Comprehensive consideration of client’s safety in terms of both the set-up of the exercise and the instructions |  |  |
| *Instructions* | Minimal or unclear instructions | Satisfactory instructions including all important elements of exercise prescription | Clear and easy to follow instructions with the inclusion of pictures. All elements of dosage clear. Font/ handwriting appropriate size and spacing |  |  |
|  |  |  |  |  | ***Total Score: /18*18** |

| Item | 0 | 1 | 2 | 3 | 4 |
| --- | --- | --- | --- | --- | --- |
| ***Goal*** | No or unsatisfactory goal provided | Satisfactory goal provided relating to improving balance | Comprehensive goal provided relating to improving balance in given client scenario |  |  |
| ***Frequency/Time*** | Frequency or time not described | Frequency or time do not meet guidelines for balance/postural control exercises | Satisfactory frequency and/or time  (at least 2 of the following:  15-20 reps, 3-5 sets, multiple times a day) | Optimal frequency and time  (15-20 reps, 3-5 sets, multiple times a day) |  |
| ***Intensity*** | Intensity/difficulty of exercise not described in sufficient detail to assess appropriateness | Intensity/difficulty of exercise not appropriate for improving hypothetical client’s balance | Exercise minimises upper limb support but may not challenge movement (amplitude/speed) of centre of mass, or base of support appropriately | Exercise minimises upper limb support and challenges at least **one** of:  - movement (amplitude/speed) of centre of mass  - base of support |  |
| ***Type*** | Exercise not appropriate to improve balance | Exercise targets standing balance but does not address removal of visual input | Exercise specifically targets postural sway with removal of visual input |  |  |
| ***Progression*** | No progressions provided | Inappropriate progression or insufficient detail provided | Acceptable progression(s) provided but may not be optimal choice **OR** guidance of when to progress not provided or not appropriate | Appropriate progression(s) with guidance on when to progress but room for improvement in some aspect of the progression, instructions or clarity. | Comprehensive progression(s) with qualitative guidance on when to progress  (e.g., able to do exercise confidently and without wobbling) |
| ***Safety*** | Client’s safety not considered (exercise set-up or instructions) | Satisfactory consideration of client’s safety (exercise set-up or instructions) | Comprehensive consideration of client’s safety in terms of both the exercise set-up and instructions |  |  |
| ***Instructions*** | Minimal or unclear instructions | Satisfactory instructions including all important elements of exercise prescription | Clear and easy to follow instructions with the inclusion of pictures. All elements of dosage clear. Font/ handwriting appropriate size and spacing. |  |  |

***Total Score: /18*18**

**BALANCE EXERCISE RUBRIC**

**Supplementary File 5 – Sensitivity analyses of pre- to post-course changes**

**in strength and balance exercise prescription quality**

| **Exercise Type** | **Pre-course Mean % (SD)**  **n=59** | **Post-course Mean % (SD)**  **n=59** | **Mean change %**  **(97.5% CI)** |
| --- | --- | --- | --- |
| Strength | 61.4 (17.9) | 69.9 (20.6) | 8.5 (6.0-10.9) |
| Balance | 55.5 (16.1) | 62.2 (17.3) | 6.8 (3.2-10.3) |

The sensitivity analysis included 4 additional participants who made no changes

to their exercise prescription tasks, resulting in a total of 59 participants

**Supplementary File 6. Percentage of participants whose exercise prescription improved, remained unchanged or regressed across rubric domains after participation in the online course**
